# Clinicopathological and genetic characteristics of pulmonary large cell carcinoma under 2015 WHO classification: a pilot study

**DOI:** 10.18632/oncotarget.21736

**Published:** 2017-10-11

**Authors:** Renwang Liu, Jinghao Liu, Tao Shi, Xiongfei Li, Dian Ren, Gang Chen, Ying Li, Hongyu Liu, Song Xu, Jun Chen

**Affiliations:** ^1^ Department of Lung Cancer Surgery, Tianjin Medical University General Hospital, Tianjin 300052, China; ^2^ Tianjin Key Laboratory of Lung Cancer Metastasis and Tumor Microenvironment, Lung Cancer Institute, Tianjin Medical University General Hospital, Tianjin 300052, China; ^3^ Department of Pathology, Tianjin Medical University General Hospital, Tianjin 300052, China

**Keywords:** large cell carcinoma, gene mutation, TP53, classification

## Abstract

Pulmonary large cell carcinoma (LCC) was re-defined under the 2015 WHO classification criteria. However, the clinicopathological features and genetic mutation statuses of Chinese LCC patients based on the new classification have rarely been investigated. Twenty-four Chinese surgically resected LCC patients previously diagnosed under the 2004 WHO criteria were re-classified under the 2015 WHO criteria. Genetic analysis was performed using next-generation sequencing of 46 cancer-related genes. The correlation of clinicopathological and genetic data was further analyzed. Eight patients were re-defined as LCCs, and 16 patients were defined as non-LCCs under the refined criteria. All LCC patients were male, and 7 patients were smokers. No significant differences in age, gender, smoking status, primary site, TNM staging and overall survival were observed between the LCC and non-LCC patients under the refined criteria. Four of the 8 LCC patients presented TP53 mutations, and no somatic mutations were detected in the other 4 LCCs under the refined criteria. For the 16 non-LCCs, not only TP53 and KRAS but also EGFR, KIT, PIK3CA, PTEN, IDH1, APC, ATM and BRAF mutations were also observed. In addition, LCCs without TP53 mutations did not present any gene mutations under the 2004 or 2015 WHO criteria. Importantly, the patients with TP53 mutation exhibited a trend with a worse survival outcome at the time of follow-up. The new WHO diagnosis criteria have superior performance in precise molecular classification for LCC patients.

## INTRODUCTION

According to the current 2015 WHO classification, pulmonary large-cell carcinoma (LCC) is morphologically defined as an undifferentiated lung carcinoma lacking the features of adenocarcinoma, squamous, small-cell carcinoma and neuroendocrine carcinoma, which can only be diagnosed from surgically resected specimens [1]. Reflecting different clinical and biological characteristics, large cell neuroendocrine carcinoma (LCNEC), basaloid carcinoma, lymphoepithelioma-like carcinoma, clear cell carcinoma and the rhabdoid phenotype, which were grouped in LCC under the previous 2004 classification, are no longer considered LCC subgroups [1].

The updated classification highlights the importance of the expanded use of immunohistochemical (IHC) staining to differentiate lung cancers lacking routine histological features. NSCLC with immunopositivity for LCNEC markers (CD56, Chromogranin or Synaptophysin), adenocarcinoma markers (TTF-1 or NapsinA) or squamous cell carcinoma markers (p40, p63 or CK5/6) is excluded under the current criteria and now reclassified as either a neuroendocrine tumor, adenocarcinoma with solid pattern or nonkeratinizing squamous cell carcinoma, respectively, according to the new 2015 WHO classification [1, 2]. NSCLC with negative immunomarkers that cannot be grouped as neuroendocrine carcinoma, adenocarcinoma or squamous cell carcinoma has now been classified as LCC with null immunohistochemical features (LCC-N).

Although LCC with adenocarcinoma, squamous cell and neuroendocrine features is excluded according to the 2015 WHO criteria, LCC-N might still represent a clinicopathologically heterogeneous entity. This finding has emphasized the importance to further classify LCC into more specific pathologic subtypes based on histological and genetic characteristics, contributing to a more accurate prognosis that leads to more personalized cancer care. Few reports have investigated the genetic status of Caucasian LCC patients under the 2015 WHO classification [3]. Driver gene profiling of Asian lung adenocarcinoma patients is markedly different from the Caucasian patients, particularly with respect to EGFR mutation status. Therefore, it is necessary to analyze the clinicopathological and genetic features in Chinese patients with LCC using the 2015 WHO classification, to explore the correlation of genotype and prognosis and to determine potential target therapeutic strategies.

## RESULTS

### Clinicopathological features and reclassification

The clinicopathological features of all 24 surgically resected LCC patients (LCC according to the 2004 WHO criteria) are summarized in Table 1. The mean age at the time of surgery was 60.33 ± 9.17 years. Twenty-three of the 24 patients were male, and 18 patients presented with a smoking history. Pathological staging was based on the 7th UICC TNM classification of NSCLC with a majority of stage III (stage I vs stage II vs stage III = 6 vs 4 vs 14). No stage IV patients were included in this cohort since surgical therapy was not recommended for these patients according the NCCN guidelines for NSCLC.

**Table 1:** Clinicopathological features of LCC under the 2004 and 2015 WHO criteria

Characteristic		Basic Information of All Specimens (*n* = 24)	Clinical Features LCC under WHO 2015 Criteria (After Reclassification via Immunohistochemistry Staining)		
			LCC ^*^(*n* = 8)	Non-LCC^#^ (*n* = 16)	*P*^
Age (y)		60.33 ± 9.17	59.88 ± 7.51	60.56 ± 10.12	0.722
Gender	Male	23 (95.8%)	8 (100.0%)	15 (93.8%)	0.480
Female		1 (4.2%)	0 (0.0%)	1 (6.3%)	
Smoke Status	Never	6 (25.0%)	1 (12.5%)	5 (31.3%)	0.328
Ever		18 (75.0%)	7 (87.5%)	11 (68.8%)	
Primary Site	Left Side	9 (37.5%)	3 (37.5%)	6 (37.5%)	1.000
Right Side		15 (62.5%)	5 (62.5%)	10 (62.5%)	
TNM staging	I	6 (25.0%)	3 (37.5%)	3 (18.8%)	0.343
	II	4 (16.7%)	2 (25.0%)	2 (12.5%)	
	III	14 (58.3%)	3 (37.5%)	11 (68.8%)	
	IV	N/A	N/A	N/A	

^*^8 of 24 patients were defined as LCC according to the 2015 WHO classification of lung cancer.

^#^Rest specimens were excluded after reclassification according to the 2015 WHO classification of lung cancer.

^*Fisher’s* exact test was selected in the condition of *n* < 40 or any *T* < 1 for 2^*^2 table *X*^2^ test.

**Figure 1:**
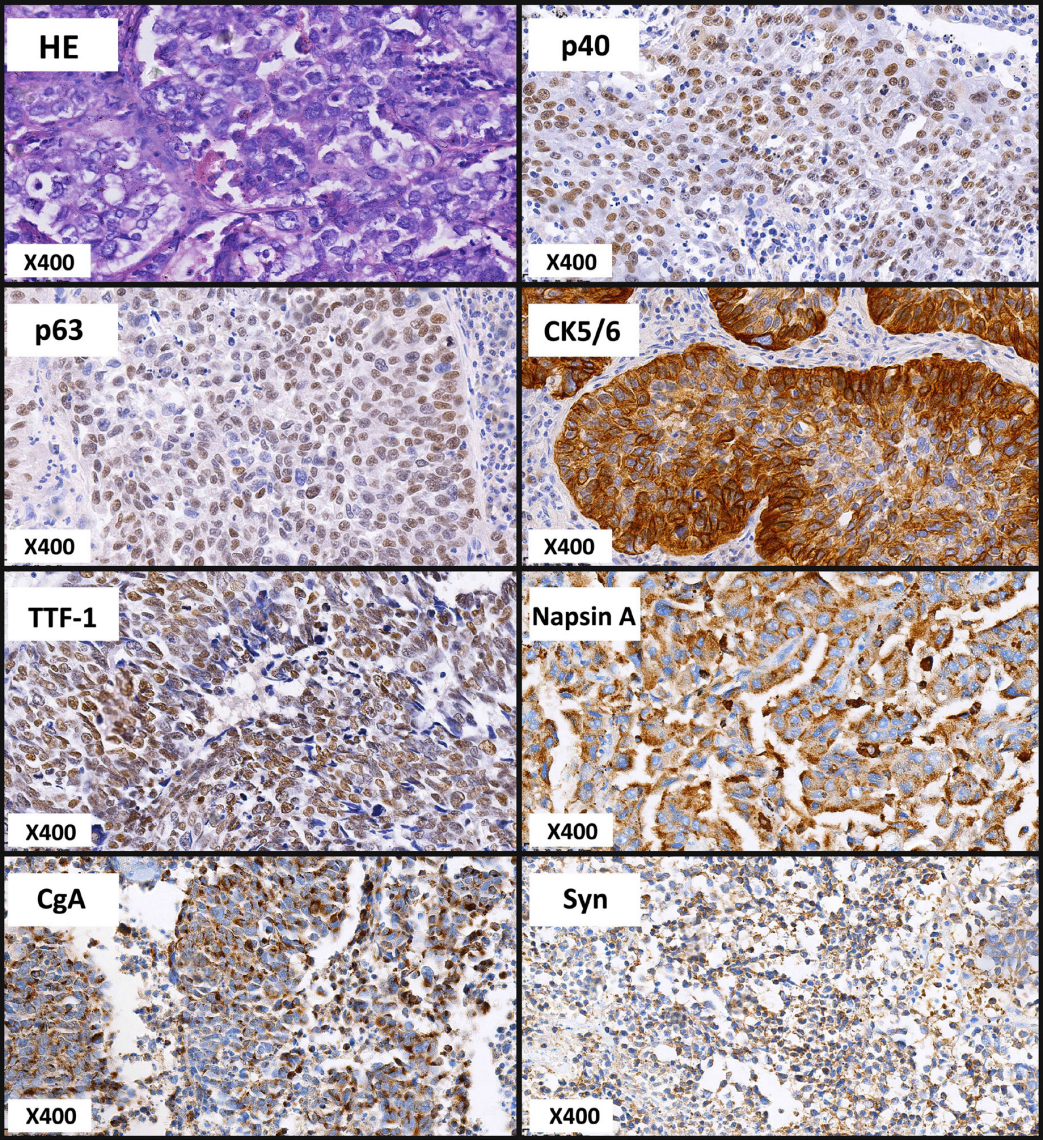
Representative images of HE and IHC staining for LCCs Immunohistochemical (IHC) staining for thyroid transcription factor-1 (TTF-1), napsin A, chromogranin A (CgA), synaptophysin (Syn), p40, p63 and CK5/6 was performed for the reclassification of all specimens. Representative images for each maker and typical HE staining were presented.

IHC staining for TTF-1, Napsin A, CgA, Syn, p40, p63 and CK5/6 was performed in each specimen to reclassify the LCC in this cohort. Eight of the 24 specimens analyzed were negative for these immunomarkers and were defined as LCC according to the 2015 WHO criteria (Table 2). Representative samples with positivity for each marker and typical HE staining of LCCs were shown in Figure 1, and the details of the IHC results for each specimen are shown in Table 2. Among the 24 patients included in the present study, no significant difference in terms of age, gender, smoking status, primary site and TNM staging (Table 1) was observed between the eight LCC and sixteen non-LCC patients, who were reclassified according to the 2015 WHO criteria.

**Table 2:** Reclassification of LCCs under the 2015 WHO criteria for lung cancer based on IHC stainin

Case No.	TTF-1	Napsin A	CgA	Syn	*p*40	*p*63	CK5/6	Reclassification of LCCs under WHO 2015 Criteria^#^
01	-	-	-	-	3+	-	-	Excluded
02^*^	-	-	-	-	-	-	-	Confirmed
03	-	-	-	-	3+	4+	3+	Excluded
04	2+	-	-	-	-	-	-	Excluded
05^	-	-	2+	2+	-	-	-	Excluded
06	2+	-	1+	-	-	-	2+	Excluded
07	-	1+	-	-	5+	-	4+	Excluded
08	4+	5+	-	-	-	-	-	Excluded
09^*^	-	-	-	-	-	-	-	Confirmed
10	-	-	-	-	1+	-	2+	Excluded
11	-	-	-	-	2+	-	2+	Excluded
12	-	-	1+	-	-	2+	-	Excluded
13	2+	-	1+	3+	-	-	-	Excluded
14	-	-	-	-	5+	5+	5+	Excluded
15^*^	-	-	-	-	-	-	-	Confirmed
16^*^	-	-	-	-	-	-	-	Confirmed
17^*^	-	-	-	-	-	-	-	Confirmed
18	4+	-	-	-	-	-	-	Excluded
19^*^	-	-	-	-	-	-	-	Confirmed
20^*^	-	-	-	-	-	-	1+	Confirmed
21^*^	-	-	-	-	-	-	-	Confirmed
22	4+	-	1+	-	-	-	-	Excluded
23	5+	-	-	5+	-	-	-	Excluded
24	5+	-	4+	4+	-	-	-	Excluded

Abbreviations: -, negative; 1+, positive tumor cells proportion range 1%–10%; 2+, positive tumor cells proportion range 11%–25%; 3+, positive tumor cells proportion range 26%–50%; 4+, positive tumor cells proportion range 51%–75%; 5+, positive tumor cells proportion range 76%–100%; LCC, large cell carcinoma; TTF-1, Thyroid transcription factor-1; CgA, chromogranin A; Syn, synaptophysin

^#^Any focal TTF-1/napsin A(≥ 1 +) or diffuse p40/p63/CK5/6 (≥ 2 +) was excluded from LCC under WHO 2015 criteria.

^Case 05 was excluded from LCC under WHO 2015 criteria and diagnosis of large cell neuroendocrine carcinoma of lung was confirmed as CgA 2+ plus Syn 2+.

^*^8 cases (case number 02, 09, 15, 16, 17, 19, 20, 21) in this cohort were confirmed as LCC under WHO 2015 criteria.

### Gene mutation profiling of LCC

**Figure 2:**
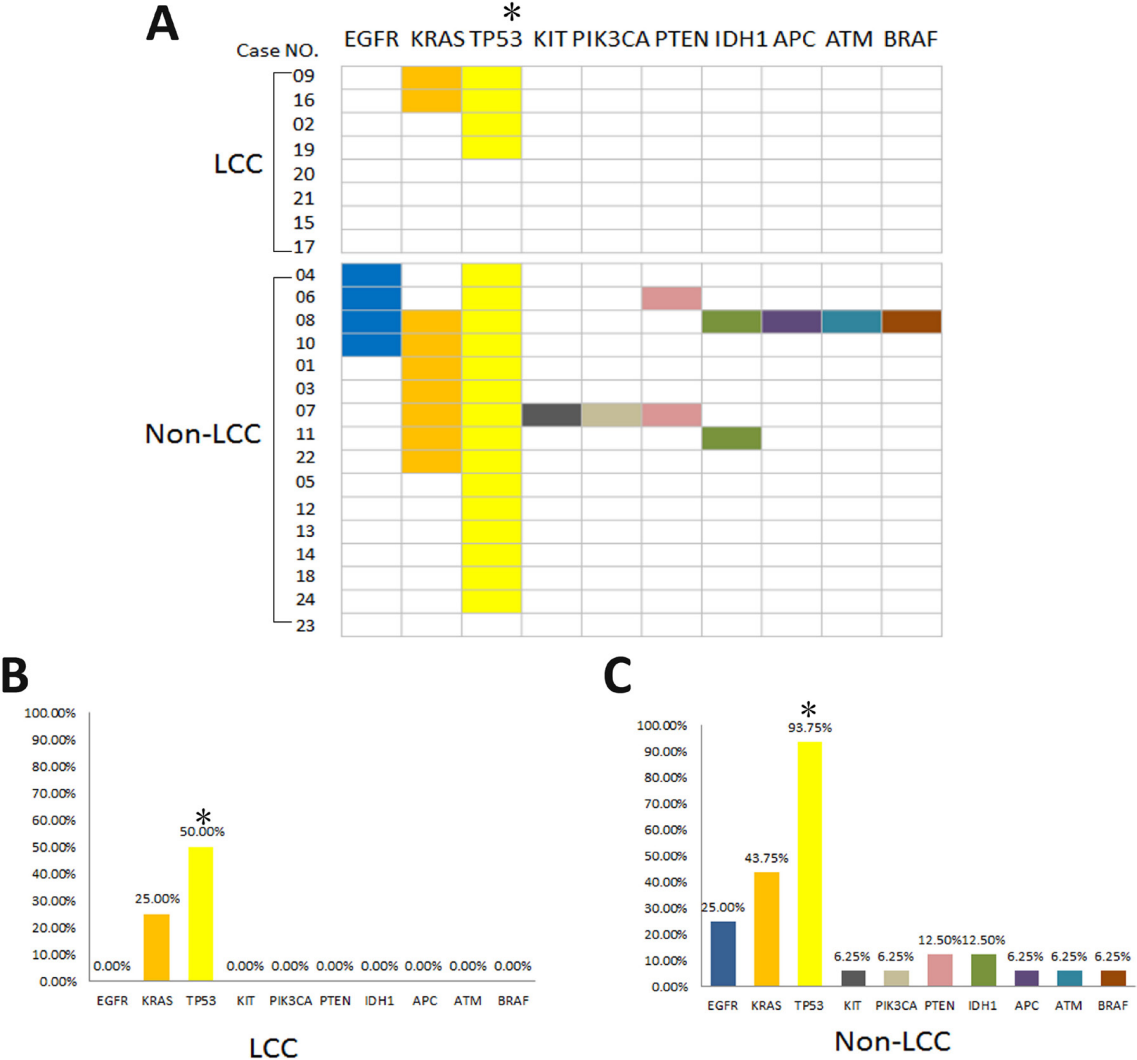
Distribution of somatic mutations for LCC and non-LCC patients under the 2015 WHO criteria (**A**) Four of the 8 LCC patients presented TP53 mutations (two patients showed concurrent KRAS mutations), and no somatic mutations were detected in the other 4 LCCs under the 2015 WHO criteria. For the 16 non-LCCs under the 2015 WHO criteria, mutations with not only TP53 and KRAS but also EGFR, KIT, PIK3CA, PTEN, IDH1, APC, ATM and BRAF were observed. (**B**) and (**C**)The percentage of each detected gene mutation in LCCs and non-LCCs under the 2015 WHO criteria. ^*^LCC patients presented a significantly lower incidence of TP53 mutation compared non-LCCs under the 2015 WHO criteria (LCC vs non-LCC = 50% vs 93.75%, respectively, *p* = 0.015).

The eight reclassified LCC samples were analyzed using next generation sequencing (NGS) to detect somatic mutations. Ten of the 46 candidate genes, including EGFR, KRAS, TP53, KIT, PIK3CA, PTEN, IDH1, APC, ATM and BRAF, were mutated in this cohort (Figure 2A). The mutated genes detected in LCC patients under the 2015 WHO criteria included TP53 (50.00%) and KRAS (25.00%) (Figure 2B). Among the eight LCC patients under the 2015 WHO criteria, four LCC patients presented TP53 (50%) mutations, and two patients presented concurrent TP53 and KRAS mutations. The other four LCC patients did not present any somatic mutations of the candidate genes analyzed (Figure 2A).

The results of gene mutation profiling were significantly different between the sixteen non-LCC specimens and eight LCC specimens according to the 2015 WHO criteria. The mutated genes with high frequency in non-LCC patients included TP53 (93.75%), KRAS (43.75%) and EGFR (25.00%) (Figure 2C). Six non-LCC patients presented single TP53 mutations, and nine non-LCC patients presented concurrent mutations in TP53 and other genes (Figure 2A). LCCs under the 2015 WHO criteria showed lower heterogeneity among the detected mutations of LCCs vs the rest = 2/46 vs 10/46, respectively, *P* = 0.030. Moreover, the reclassified LCC patients presented a significantly lower incidence of TP53 mutation compared with the excluded specimens according to the 2015 WHO criteria (LCC vs non-LCC = 50% vs 93.75%, respectively, *p* = 0.015).

### Gene mutations and LCC clinical characteristics

**Figure 3:**
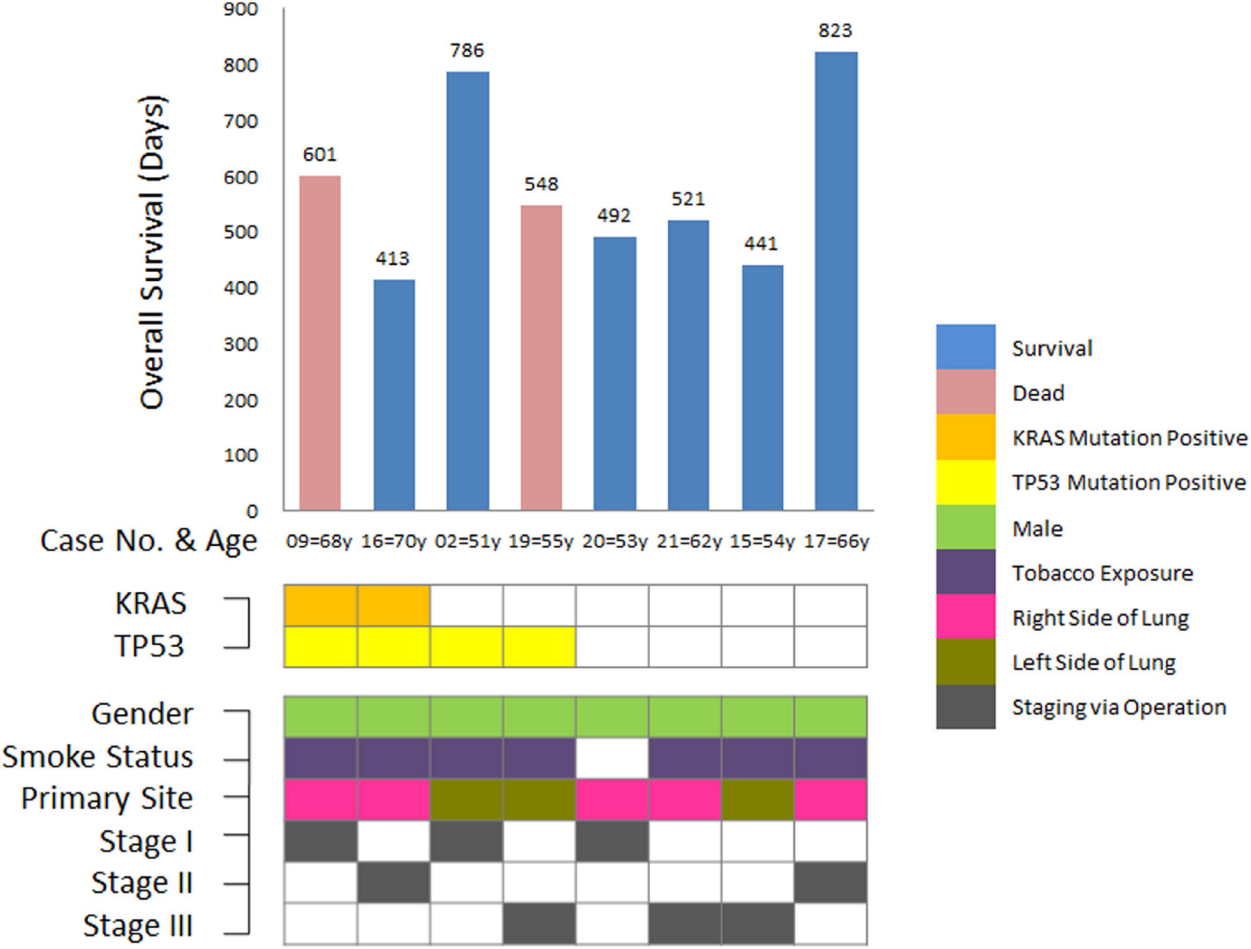
Somatic mutations and clinical features of LCC patients under the 2015 WHO criteria Only TP53 and KRAS mutations were detected in all 8 LCC patients under the 2015 WHO classification. Concurrent mutations in TP53 and KRAS were observed in 2 LCC patients. The mean survival time for all LCC patients under the 2015 WHO criteria was 698.75 ± 62.83 days. All LCC patients were male, and 7 patients were smokers. Three of the 8 tumors primarily occurred in the left side of the lungs. The TNM staging distribution was shown as I/II/III = 3/2/3.

None of the somatic mutations, except KRAS and TP53, were detected in all 8 lung large cell carcinoma under the 2015 WHO classification of NSCLC. The distribution and specific clinical features of these mutations are shown in Figure 3. All 8 patients in this group were male, and only one patient was a non-smoker who did not present any gene mutation. Five patients had right lesions, and three patients had left lesions. The staging of the 8 LCC patients is shown in Figure 3. The survival time and status of each patient redefined as LCC according to the 2015 WHO criteria are also shown in Figure 3. Two patients (Case No.09: with KRAS plus TP53 mutations, Stage I; Case No.19: with a single TP53 mutation, Stage III) died at 601 days and 548 days after operation, respectively. The mean survival time of all 8 patients was 698.75 ± 62.83 days.

### Overall survival analysis for LCCs under the 2015 WHO criteria

**Figure 4:**
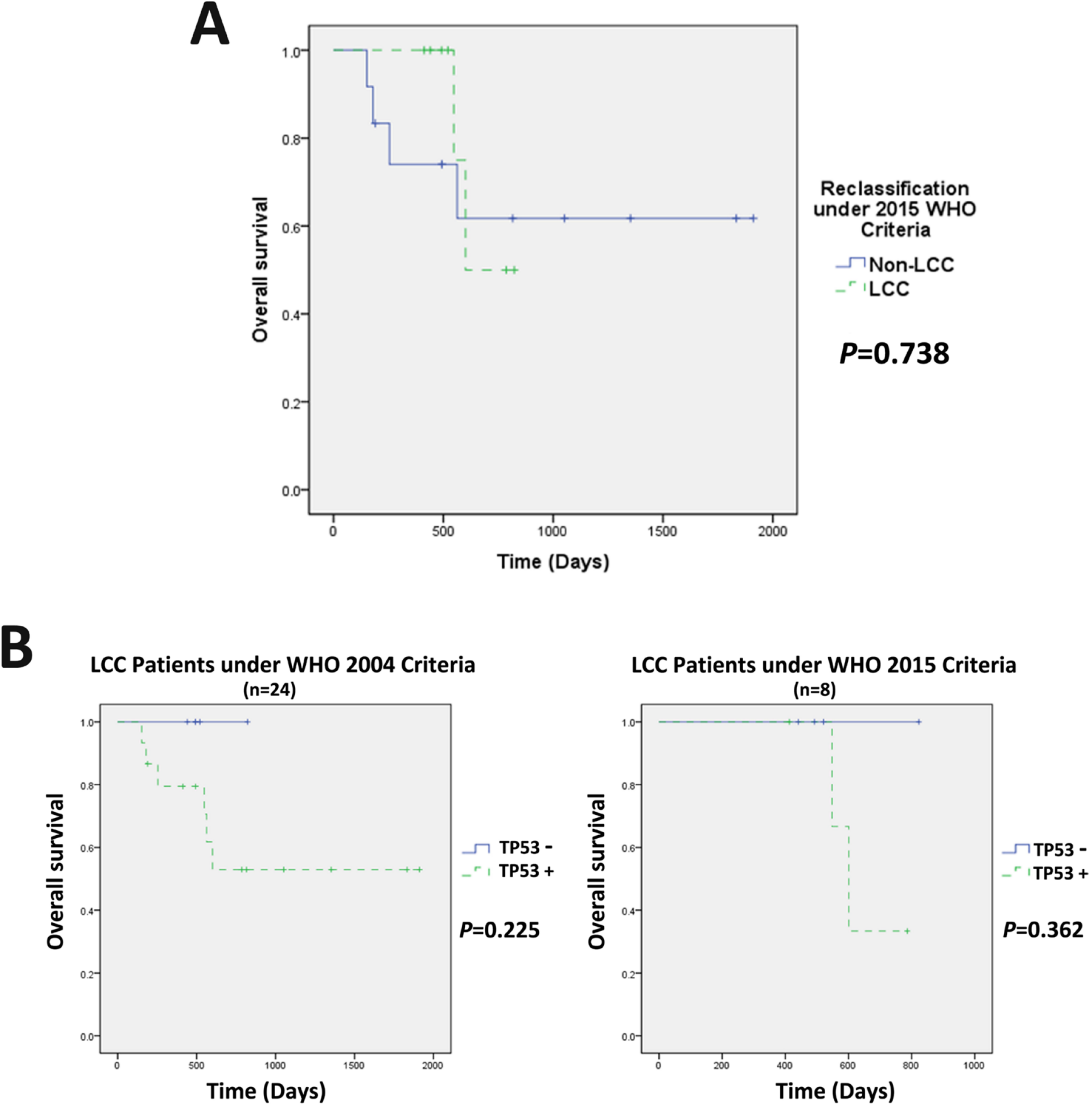
Overall survival analysis for LCCs under the 2015 WHO criteria (**A**) Twenty-four LCC patients previously diagnosed under the 2004 WHO criteria were re-classified according to the 2015 WHO criteria. The mean survival time of LCC vs non-LCC patients under the 2015 WHO criteria was 698.75 ± 62.83 vs 1301.03 ± 245.40 (days), respectively, *p* = 0.738; (**B**) LCCs without TP53 mutations did not present any gene mutations under the 2004 or 2015 WHO criteria. Interestingly, patients without TP53 mutations showed better survival outcomes at the time of follow-up, although without significant difference.

The twenty-four LCC patients previously diagnosed under the 2004 WHO criteria were re-classified according to the 2015 WHO criteria, and their survival statuses were compared. No significant difference in the overall survival time was observed between the eight LCC and sixteen non-LCC patients according to the 2015 WHO criteria (LCC vs non-LCC = 698.75 ± 62.83 d vs 1301.03 ± 245.40 d, respectively, *P* = 0.738) (Figure 4A). In addition, we investigated the correlation between survival and TP53 mutation in LCC patients defined according to the 2004 or 2015 WHO classification criteria (Figure 4B). Five patients without TP53 mutations, including four patients reclassified as LCCs and one non-LCC patient according to the 2015 WHO criteria, were absent of any other mutation (Figure 2A) and were alive at the time of follow-up. The patients with TP53 mutations, under both the 2004 and 2015 WHO criteria, showed shorter survival, although without significant difference, probably due to the limited patient number.

## DISCUSSION

According to the 2015 WHO classification, the definition of pulmonary LCC was described as an undifferentiated NSCLC that lacks the cytologic, architectural, and immunohistochemical features of small cell carcinoma, adenocarcinoma or squamous cell carcinoma. In the new criteria, the effect of immunohistochemical feature is particularly emphasized in comparison with the WHO 2004 classification which defined LCC based on the architectural characters [4, 5]. This update in the classification of lung cancer leads to an enormous change in the histological type of pulmonary LCC. Tumors with features of LCNEC or focal positive IHC staining for TTF-1/NapsinA or diffuse positive IHC staining for p40/p63/CK5/6 are excluded from LCC and are now classified as solid adenocarcinoma or non-keratinizing squamous compared to the WHO 2004 classification [2]. Treatment regimens for LCC with new classification have not been updated and the previous strategies are still recommended currently [6–8]. Whether the revised the refined classification of LCC could influence the therapy decision needs further investigation. The study of LCC under the new criteria is extremely rare. Previous studies have attempted to redefine LCC as a marker-negative or marker-null large cell carcinoma with the absence of TTF-1 and p40 based on IHC staining. *Natasha Rekhtman* and colleagues demonstrated that most patients with marker-null LCC were male smokers after reclassifying 102 patients diagnosed with LCCs according to the 2004 WHO criteria [9]. In our study, we also observed that under the 2004 or 2015 WHO criteria a large proportion of LCC patients were male and had tobacco exposure. However, the LCC patients showed similar clinicopathological features, including age, gender, smoking status, primary site and TNM staging, compared to the excluded patients under the 2015 WHO criteria (Table 1).

Recent studies have demonstrated that marker-null patients tended to have a worse response to therapy and inferior OS in NSCLC [10–12]. *Natasha Rekhtman* et al. indicated that LCC patients with marker-null phenotypes showed inferior disease-free survival (DFS) and OS compared to other patients with adenocarcinoma or squamous immunoprofiles [9]. *Anna Karlsson* et al. reclassified LCC patients diagnosed under the 2004 criteria and observed that marker-negative LCCs had an inferior OS compared to patients with positive markers for TTF-1/Napsin A [13]. Thus, LCCs under the 2015 WHO criteria might have worse prognoses, as most LCCs presented no IHC phenotype. In the present study, we did not observe a significant difference of overall survival time between LCC and non-LCC patients under the 2015 WHO criteria (Figure 4A). However, due to the limited cases in our study, further verification with larger samples is very necessary.

Previous studies focusing on the genetic profile of LCCs according to the 2015 WHO classification have rarely been reported. Under the 2004 WHO criteria, the most frequent mutations in LCCs occurred in TP53 and KRAS genes, consistent with our findings [9, 13–15]. LCCs with IHC marker-null phenotypes were also associated with a high incidence of TP53 and KRAS mutations [9, 15]. The high incidence of TP53 mutations in many pulmonary tumors and the correlation of KRAS mutations with smoking status have been reported [16, 17]. In the present study, both LCC and non-LCC patients under the 2015 WHO criteria presented a high incidence of TP53 (50% vs 93.75%, respectively) and KRAS (25% vs 43.75%, respectively) mutations (Figure 2). Under the 2015 WHO criteria, we also observed that LCC patients only presented TP53 and KRAS mutations in the 46 gene panel, while non-LCC patients showed 8 additional mutations in genes other than TP53 and KRAS (LCC vs non-LCC = 2/46 vs 10/46, respectively). These data indicate that the molecular phenotype of LCCs under the 2015 WHO criteria is more homogeneous compared to the old criteria. Furthermore, although there was no significant difference, all four LCC patients under the 2015 WHO criteria who did not harbor TP53 mutations were alive at the time of follow-up and might present better prognoses compared with the other patients in this cohort. Interestingly, all of four patients did not harbor any other mutations. *Giuseppe Pelosi* et al. reported that LCC patients with less than 3 gene mutations had a better PFS and OS outcome compared with patients harboring 3 or more than 3 mutations [15]. Taken together, these results suggest that LCCs with fewer genetic mutations might present better prognoses.

Recently, *Brandon* et al. reported the genetic profile of LCC reclassified according to the 2015 WHO criteria [3]. Only two of 17 cases were reclassified as LCC under the 2015 WHO criteria, reflecting the rare incidence of LCCs. These authors observed that adenocarcinoma with solid pattern (ADC-S) and nonkeratinizing squamous cell carcinoma (NK-SQCC), which were excluded from the 2015 WHO criteria of LCC, exhibited various gene mutations, including EGFR, KRAS, TP53 and other genes. The two ADC-S and one NK-SQCC patients also presented concurrent mutations, while no concurrent mutations were detected in large cell carcinoma with null immunohistochemical features (LCC-N). These results indicated that LCCs under the 2015 WHO criteria may have a more homogeneous genotype. In addition, although the statistical analysis was not provided, the previous study reported that the incidence of TP53 mutations of LCC-N was lower than ADC-S and NK-SQCC (LCC-N vs ADC-S + NK-SQCC = 0/2 vs 7/15, respectively), consistent with the results of our study. However, a discrepancy concerning TP53 and KRAS mutations in LCCs under the latest criteria was observed between our study and that of *Brandon* et al., likely reflecting the limited number of LCC specimens in both studies.

Previous studies have demonstrated that EGFR mutations are strongly correlated with clinical features, including Asian, female, non-smokers and adenocarcinoma [18–20]. The rare incidence of EGFR mutations was reported in LCCs under the 2004 WHO criteria and LCCs with IHC marker-null phenotypes [9, 13–15]. In our study, no EGFR mutations were observed under the 2015 WHO classification, while four of the sixteen excluded tumors harbored EGFR mutations (Figure 2). Interestingly, three of the four patients were redefined as adenocarcinoma or adenosquamous cell carcinoma and one patient was redefined as squamous cell carcinoma according to the 2015 WHO criteria because of positive TTF-1 and CK5/6 IHC staining, respectively (Table 2). Moreover, we did not observe the incidence of anaplastic lymphoma kinase (ALK) arrangement in LCCs under the 2004 or 2015 WHO criteria.

There are some limitations for our study. Firstly, this is a retrospective analysis and some confound variables are not provided. Secondly, due to the low prevalence, the number of LCC, especially for LCC with refined classification, is small. Thirdly, the LCC patients recruited in this study are Chinese race. The difference of LCC patients from Chinese and western race can not be directly compared. These factors may lead to a bias of the results. Multi-center randomized controlled trials with larger samples are necessary to further verify our findings.

In conclusion, we reclassified Chinese surgically resected LCC patients according to the 2015 WHO classification of lung cancer. Consistent with the 2004 WHO criteria, under the 2015 WHO criteria, a large proportion of LCC patients were male and had tobacco exposure. No significant difference in terms of age, gender, smoking habits, primary site and TNM staging were observed between LCCs and excluded patients according to the 2015 WHO criteria. LCCs with refined classification exhibit more homogeneous in the aspects of immuno- and genetic phenotypes. TP53 and KRAS were the most frequent gene mutations for LCCs under the 2015 WHO criteria. No other somatic gene mutations, including EGFR and ALK, were detected in our study. Surgically resected LCCs with TP53 mutations showed a worse survival tendency, while LCCs without any detected mutations might indicate a better prognosis.

## MATERIALS AND METHODS

### Patients and reclassification

The present study included surgical pathology blocks of twenty-four LCC patients who were diagnosed according to the 2004 WHO criteria between 2009–2013 at Tianjin Medical University General Hospital. The collected samples were retrospectively reviewed and reclassified according to the 2015 WHO criteria. All patients received radical resection via open or minimally invasive thoracic surgery, followed by four cycles of adjuvant chemotherapy. This study was approved by the institutional review board of Tianjin Medical University General Hospital. The study was conducted in compliance with the ethical principles of the Declaration of Helsinki. All patients provided written consent.

HE staining and specific IHC staining was repeated on whole tissue sections from the blocks for the reclassification of LCC. Each tumor was re-examined by 8 samples as one for HE staining and seven for seven different IHC markers. The specimens of positive immunophenotype, including LCNEC markers (Chromogranin or Synaptophysin), adenocarcinoma markers (TTF-1 or NapsinA) and squamous cell carcinoma markers (p40, p63 or CK5/6), were excluded from LCC according to the 2015 WHO criteria. All antibodies used in this study were purchased from ZSGB-BIO, Beijing, China. Specimens with more than 1% positivity for TTF-1/napsin A and 10% positivity for p40/p63/CK5/6 expression were excluded from LCCs under the 2015 WHO criteria in this study. Specimens with CgA and Syn positivity, a feature of neuroendocrine tumors, were also excluded. The reclassification was reviewed by two pathologists independently.

### Next generation sequencing

DNA preparation and sequencing was performed at San Valley Biotechnology Incorporated, Beijing, China. Total DNA was extracted from each formalin-fixed, paraffin-embedded (FFPE) specimen using the QIAamp DNA Mini Kit (QIAGEN). An Ion Torrent adapter-ligated library was constructed using the Ion AmpliSeq Library Kit 2.0 (Life Technologies) and purified using AMPure beads (Beckman Coulter). Emulsion PCR was performed using an IKADT-20 mixer (Life Technologies) after adding Ion Sphere Particles (ISPs). The enrichment of template-positive ISPs was performed using Dynabeads MyOne Streptavidin C1 beads (Life Technologies) and confirmed using a Qubit 2.0 fluorometer (Life Technologies). Sequencing reactions were subsequently performed using the Ion PGM 200 Sequencing Kit (Life Technologies). Forty-six mutations, including EGFR, BRAF, TP53, ALK and other cancer-related gene mutations, were targeted in this study (Table 3).

**Table 3:** Next generation sequencing for mutations in 46 cancer-related genes

ABL1	AKT1	ALK	APC	ATM	BRAF	CDH1
CDKN2A	CSF1R	CTNNB1	EGFR	ERBB2	ERBB4	FBXW7
FGFR1	FGFR2	FGFR3	FLT3	GNAS	HNF1A	HRAS
IDH1	IDH2	JAK3	KDR	KIT	KRAS	MET
MLH1	MPL	NOTCH1	NPM1	NRAS	PDGFRA	PIK3CA
PTEN	PTPN11	RB1	RET	SMAD4	SMARCB1	SMO
SRC	STK11	TP53	VHL			

### Statistical analysis

All variables were analyzed using *IBM SPSS Statistics 22.0 for Windows*. The Independent-samples T test was used to analyze the measurement data and *X*^2^ test count data. *Fisher’s* exact test was used under conditions of *n* < 40 or any T < 1, and *Yates’* correction for continuity was used under conditions of *n* ≥ 40, 1 ≤ any T ≤ 5 for the 2*2 table X^2^ test. Survival analyses were performed using the Kaplan-Meier method with the log-rank test. *P* < 0.05 was set as a statistically significant difference.
